# Do cravings predict smoking cessation in smokers calling a national quit line: secondary analyses from a randomised trial for the utility of ‘urges to smoke’ measures

**DOI:** 10.1186/s13011-015-0011-8

**Published:** 2015-04-14

**Authors:** Jaspal S Taggar, Sarah Lewis, Graeme Docherty, Linda Bauld, Andy McEwen, Tim Coleman

**Affiliations:** Division of Primary Care, University of Nottingham, Medical School, Queen’s Medical Centre, Nottingham, NG7 2UH UK; Division of Epidemiology & Public Health, University of Nottingham, Nottingham, NG7 2UH UK; Institute for Social Marketing, University of Stirling, Stirling, UK; Cancer Research UK Health Behavioural Research Centre, University College London, London, UK

**Keywords:** Urges to smoke, Smoking cessation, Heaviness of smoking index

## Abstract

**Background:**

Single-item urges to smoke measures have been contemplated as important measures of nicotine dependence This study aimed to prospectively determine the relationships between measures of craving to smoke and smoking cessation, and compare their ability to predict cessation with the Heaviness of Smoking Index, an established measure of nicotine dependence.

**Methods:**

We conducted a secondary analysis of data from the randomised controlled PORTSSS trial. Measures of nicotine dependence, ascertained before making a quit attempt, were the HSI, frequency of urges to smoke (FUTS) and strength of urges to smoke (SUTS). Self-reported abstinence at six months after quitting was the primary outcome measure. Multivariate logistic regression and Receiver Operating Characteristic (ROC) analysis were used to assess associations and abilities of the nicotine dependence measures to predict smoking cessation.

**Results:**

Of 2,535 participants, 53.5% were female; the median (Interquartile range) age was 38 (28–50) years. Both FUTS and HSI were inversely associated with abstinence six months after quitting; for each point increase in HSI score, participants were 16% less likely to have stopped smoking (OR 0.84, 95% C.I 0.78-0.89, p < 0.0001). Compared to participants with the lowest possible FUTS scores, those with greater scores had generally lower odds of cessation (p across frequency of urges categories=0.0026). SUTS was not associated with smoking cessation. ROC analysis suggested the HSI and FUTS had similar predictive validity for cessation.

**Conclusions:**

Higher FUTS and HSI scores were inversely associated with successful smoking cessation six months after quit attempts began and both had similar validity for predicting cessation.

**Electronic supplementary material:**

The online version of this article (doi:10.1186/s13011-015-0011-8) contains supplementary material, which is available to authorized users.

## Background

A core feature of drug addiction is the difficulty in reducing or stopping use of substances [1,2]. Tobacco smoking is a major addiction which causes premature morbidity, mortality and impaired quality of life [3,4], the risks of which can be reduced by smoking cessation. However, smokers’ ability to reduce or stop smoking may be less and their risks of relapse or continued smoking may be higher when they have higher levels of dependence on cigarettes [5]. Consequently, it is important to have simple, easily-replicable and robust measures of nicotine dependence, as being able to accurately measure this crucial quality could help lead greater understanding of factors associated with successful smoking cessation or continued smoking and perhaps even lead to more efficient targeting of cessation interventions [6-8]. There is no single definition of nicotine dependence and consequently there is no universal measurement of it, however, a clinically important consideration when developing nicotine dependence measures is that they should have an ability to predict relapse back to smoking after abstinence [9].

Quantitative, biochemically-verified questionnaires are commonly used to measure nicotine dependence [10]. Two measures, the Fagerström Test for Nicotine Dependence (FTND) and its shorter derivative, the two-item Heaviness of Smoking Index (HSI), have been biochemically validated and are widely used in research and clinical practice [11,12]. These measures predominately rate smoking behaviour reported by smokers themselves and higher scores are associated with greater nicotine use and increased risk of relapse after quitting [13,11,12]. However, nicotine dependence is likely multi-faceted and subjective symptoms of nicotine dependence are also thought to be important and have been conceptualised in theories of dependence [14] such as , the Plans–Responses–Impulses–Motives–Evaluations (PRIME) theory. PRIME theory suggests that smokers experience impulses and motivation to continue smoking [15] and these impulses manifest as cravings or urges to smoke (UTS) which vary over time in frequency and intensity and may influence successful smoking cessation [15].

Greater urges or cravings have been found to be associated with an increased risk of relapse to smoking [16]. Single-item measures which investigate smokers’ experiences of nicotine withdrawal and which rate the frequency and strength of smokers’ urges to smoke (i.e. withdrawal symptoms) are brief and easy to administer [17]; recently [18,19,17], two analyses of survey data from the Smoking Toolkit Study found that, compared to the HSI, there was a stronger association between smokers’ strength of urges to smoke (SUTS) experienced during a normal smoking day and relapse up to six months after quitting [20,18,19]. However, frequency of urges to smoke (FUTS)) was not found to be associated with relapse to smoking [18,19]. In contrast, an International Tobacco Control (ITC) four-country survey investigated predictors of smoking relapse [21], and found greater FUTS one month after quitting were associated with relapse one year later [21]. Although these surveys included smokers representative of general smoking populations, there have been no prospective studies that have tested the generalizability and replicability of findings for the single-item UTS measures to predict abstinence in other smoking populations, such as motivated smokers that are seeking cessation treatment.

Using data from a smoking cessation quitline trial, we conducted secondary analyses to prospectively investigate, within motivated, treatment-seeking smokers, the relationship between the symptoms of nicotine withdrawal (i.e. cravings to smoke) and subsequent smoking cessation. In the same dataset, we investigate the relationship between HSI, a more established nicotine dependence measure based on reported smoking behaviour and compare the utility of this with symptom-based measures for predicting smoking cessation.

## Methods

### Data sources and study design

Participant data from the Proactive Or Reactive Telephone Smoking CeSsation Support (PORTSSS) trial, a factorial Randomised Controlled Trial (RCT), were used as a cohort for this study [22,23]. This tested the efficacy of proactive telephone support for smoking cessation, delivered by a publicly-funded English smoking cessation helpline, in comparison to standard telephone support, and whether or not additional free Nicotine Replacement Therapy offered via the quitline affected smoking cessation rates. The protocol, selection criteria and trial outcomes have previously been reported; briefly the trial recruited non-pregnant smokers aged 16 or more residing in England who contacted a quitline, wanted help with cessation and agreed to set a date for quitting smoking between four days and four weeks of initial contact [22,23]. A total of 2,591 participants were randomised. Consent was withdrawn by 56 participants; therefore data from 2,535 participants were used for the current analysis. The characteristics of participants used for our analyses were similar to those from the PORTSSS trial. Less than 5% missing data were reported for each baseline variable.

### Measures

#### Participant characteristics

Data for participant characteristics were collected at study recruitment and variables used in the analyses included: age (years), gender, ethnicity, Index of Multiple Deprivation (IMD) score (a UK government measure of deprivation based on income, employment, health deprivation and disability, education skills and training, barriers to housing and services, crime and living Environment; a higher composite score represents greater deprivation) [24], presence of regular smokers residing in the same household, entitlement to free prescriptions for medication, serious attempt to quit smoking made in the last 12 months, use of any smoking cessation support during the last quit attempt, current use of any smoking cessation support and study treatment allocation.

#### Independent variables

At study entry, (before making a quit attempt) we ascertained from participants’ self-reported i) frequency of urges to smoke (FUTS) in the last 24 hours [17], ii) strength of urges to smoke (SUTS) in the last 24 hours [17] and iii) the Heaviness of Smoking Index (HSI). The Urges to smoke (UTS) and HSI measures were graded on six and seven point Likert scales, respectively. FUTS was ascertained by asking participants “How much of the time have you felt the urge to smoke in the past 24 hours?” Responses were categorised as 1) not at all, 2) a little of the time, 3) some of the time, 4) a lot of the time, 5) almost all of the time and 6) all of the time. SUTS was ascertained by asking participants “How strong have the urges been in the past 24 hours?” Responses were categorised as 1) none, 2) slight, 3) moderate, 4) strong, 5) very strong and 6) extremely strong. The components of the HSI have been previously reported [12]. Responses of “don’t know” were coded as missing.

#### Dependant variables [Outcomes]

Smoking status at six months after participants’ quit date, which was set within four weeks of randomisation and collection of baseline data in the trial, was used to determine smoking cessation. For trial outcomes, in accordance with the Russell standard [25], cessation was defined as self-reported abstinence or having smoked ≤5 times in total since the quit date and smokers who were lost to follow-up (n=1,181) were assumed to still be smoking. This approach to missing data assumes that data is missing not at random, and specifically that loss to follow-up equates to continued smoking (or relapse to smoking), and is standard in smoking cessation trials. However, we tested in sensitivity analyses an approach based on an alternative assumption, as described below.

### Statistical analyses

Analyses were performed using Stata version 11.0. The prevalence or mean (SD)/median (IQR) of participant characteristics were calculated for categorical or continuous data, respectively, and compared in those with and without cessation outcomes. Participant characteristics were also compared across categories of each measure of nicotine dependence. Continuous data for participant characteristics were re-categorised into quintiles in order to compare participant characteristics and the measures of dependence. Comparisons were made using students t-test or analysis of variance (ANOVA) where participant characteristics were binary variables or consisted of more than two categories, respectively.

Prior to multivariate modelling the linearity of the nicotine dependence measures was assessed by comparing the bivariate relationship between cessation and each dependence measure fitted in categories or as a continuous variable; differences between the two models were determined using the likelihood ratio test (LRT) and nicotine dependence measures were subsequently entered into multivariate models as categorical or continuous data, as appropriate. An assessment of multi-collinearity between the FUTS, SUTS and HIS measures was made using Variance Inflation factor (VIF) analysis [26]. This provided an assessment of the degree of independent variance amongst the variables and a single VIF > 10 or tolerance (1/VIF) < 0.1 was used to indicate the presence of substantial collinearity.

Logistic regression adjusting *a priori* for age, gender and treatment group was used to determine the association between each measure of nicotine dependence and smoking cessation at six months with the primary analysis using the assumption that loss to follow up was equivalent to a participant having returned to smoking [27]. An assessment for other confounding variables (ethnicity, IMD score, smoker residing in same household, free entitlement to NHS prescriptions, quit attempt in last 12 months, cessation support used in last 12 months, cessation support used in last quit attempt and current use of cessation support) was made and, any identified, were included in the multivariate model as appropriate; a significant confounder was defined as a covariate significantly associated with both the measure of nicotine dependence and outcome, and one that altered the univariable association between these by ≥10%. To determine if the association between dependence measures and outcomes was modified according to categories of age or gender, an assessment of interaction by these variables was made using the LRT. Throughout all analyses the significance of associations was determined using the Wald test, for binary variables, or LRT for categories and trend, for categorical and ordinal variables, respectively. A p value of <0.05 indicated statistical significance. The ability of the UTS and HSI measures to predict smoking cessation was determined using Receiver Operating Characteristic (ROC) analysis [28]. The multivariate model for each statistically significant association between measure of dependence and outcome was entered separately into ROC models. An assessment of study power found the dataset had a 90% power to detect an association between the measures of nicotine dependence and smoking cessation at the 5% significance level.

To investigate the appropriateness of our assumption that those lost to follow up were still smoking, we conducted a multiple imputation sensitivity analysis based on the alternative assumption that outcome data were missing at random. We used the mi commands in Stata and all baseline variables in the imputation model (20 imputations). Logistic regression, adjusting for the same variables as in the primary analyses, was used to determine the associations between measures of dependence and smoking cessation. The statistical significance of associations was determined using the joint Wald’s test. Differences between the principal and sensitivity analyses were compared and important differences are commented on qualitatively.

## Results

### Participant characteristics and their associations with measures of nicotine dependence

Of the 2,535 participants the median (IQR) age and IMD scores were 38 (28–50) and 23.1 (13.5-37.1), respectively; 1,355 (53.5%) were female and 2,281 (90.0%) were Caucasian. The UTS and HSI measures were normally distributed; the mean (SD) FUTS, SUTS and HSI scores were 4 (1.2), 4 (1.1) and 3 (1.6), respectively. Of all participants, 469 (18.5%) reported abstinence at six months. There were 1,354 (53.4%) participants that provided data for cessation outcomes at six months. Participants lost to follow up were younger (median (IQR) age: 35 (25–46) versus 40 (30–53), Mann- Witney test p < 0.0001), experienced greater social deprivation (median (IQR) IMD score: 24 (14.1-38.3) versus 22.1 (13.0-37.7), Mann–Whitney test p=0.0382) and had greater HSI scores (mean (SD) HSI score: 3.3 (1.6) versus 3.1 (1.6), Students t-test p=0.0042; d.f 2509) than those who provided follow up data for smoking status (Additional file 1: Table S1).

The associations between participant characteristics and measures of dependence are presented in Table 1. The mean FUTS and SUTS scores were similar across quintiles of ascending age. However, the mean (SD) HIS score significantly increased from 2.9 (1.5) to 3.5 (1.5) in participants with the lowest to highest age quintile, respectively. There were no significant differences in mean scores of the dependence measures across categories of gender. Greater IMD scores were associated with significantly higher ratings of the HSI; participants in the lowest quintile of IMD score reported a mean (SD) HSI score of 2.9 (1.6) and this increased to 3.5 (1.5) in those categorised to the highest IMD quintile. Participants residing in the same household as smokers were more likely to report greater FUTS and HSI scores than those living without other smokers. Greater FUTS, SUTS and HIS scores were reported in participants entitled to free NHS prescriptions and those who reported using cessation support during their last quit attempt. The mean FUTS, SUTS and HSI scores for those entitled to free NHS prescriptions were 3.6 (1.2), 3.6 (1.2) and 3.4 (1.5), respectively. There were no significant differences in the scores of all dependence measures according to the PORTSS study treatment allocation.

**Table 1:** **Associations between participant characteristics and the measures of nicotine dependence**

**VARIABLE**	**FUTS**			**SUTS**			**HSI**		
	**Mean (s.d)**	**d.f**	**P-value**	**Mean (s.d)**	**d.f**	**P-value**	**Mean (s.d)**	**d.f**	**P-value**
Age									
Quintile 1	3.5 (1.1)	4; 2414	0.3035	3.6 (1.1)	4	0.9768	2.9 (1.5)	4	<0.01
Quintile 2	3.4 (1.1)			3.5 (1.1)			2.7 (1.6 )		
Quintile 3	3.5 (1.2)			3.6 (1.2)			3.3 (1.5)		
Quintile 4	3.6 (1.2)			3.6 (1.1)			3.6 (1.6)		
Quintile 5	3.5 (1.2)			3.6 (1.1)			3.5 (1.5)		
Gender									
Male	3.5 (1.2)	2439	0.6899	3.5 (1.1)	2439	0.2456	3.2 (1.6)	2466	0.2637
Female	3.5 (1.1)			3.6 (1.1)			3.2 (1.6)		
Ethnicity									
White	3.5 (1.2)	3; 2480	0.7406	3.6 (1.1)	3	0.7051	3.2 (1.6)	3	<0.01
Black/mixed	3.5 (1.2)			3.7 (1.2)			2.8 (1.6)		
Asian/mixed	3.5 (1.3)			3.5 (1.0)			2.7 (1.6)		
Other	3.4 (1.3)			3.7 (1.3)			3.2 (1.6)		
IMD score									
Quintile 1	3.4 (1.2)	4; 2479	0.0618	3.6 (1.2)	4	0.1203	2.9 (1.6)	4	<0.01
Quintile 2	3.5 (1.2)			3.5 (1.1)			3.0 (1.6 )		
Quintile 3	3.5 (1.1)			3.6 (1.1)			3.2 (1.6)		
Quintile 4	3.5 (1.2)			3.5 (1.1)			3.4 (1.5)		
Quintile 5	3.6 (1.2)			3.7 (1.2)			3.5 (1.5)		
Smoker in same household									
No	3.5 (1.2)			3.5 (1.1)			3.2 (1.6)		
Yes	3.6 (1.2)	2478	<0.05	3.6 (1.1)	2478	0.2063	3.3 (1.6)	2505	<0.05
Free prescription entitlement									
No	3.4 (1.1)			3.4 (1.1)			2.9 (1.6)		
Yes	3.6 (1.2)	2456	<0.01	3.6 (1.2)	2456	<0.01	3.4 (1.5)	2483	<0.01
Quit attempt in last 12 months									
No	3.5 (1.2)			3.5 (1.1)			3.2 (1.6)		
Yes	3.6 (1.1)	2476	0.0621	3.7 (1.1)	2475	<0.01	3.2 (1.6)	2501	0.2932
Cessation support last quit attempt									
No	3.5 (1.2)			3.5 (1.1)			3.1 (1.6)		
Yes	3.7 (1.1)	2482	<0.01	3.7 (1.1)	2482	<0.01	3.4 (1.5)	2509	<0.01
Current cessation support									
No	3.5 (1.2)			2.5 (1.1)			3.2 (1.6)		
Yes	3.6 (1.2)	2482	0.2969	3.6 (1.1)	2482	0.3838	3.4 (1.5)	2509	<0.01
Trial intervention									
Reactive support without NRT	3.6 (1.2)	3	0.7149	3.5 (1.2)	3	0.6796	3.2 (1.6)	3	0.9512
Reactive support with NRT	3.5 (1.1)			3.6 (1.1)			3.2 (1.6)		
Proactive support without NRT	3.5 (1.2)			3.5 (1.1)			3.2 (1.6)		
Proactive support with NRT	3.5 (1.2)			3.6 (1.1)			3.2 (1.6)		

FUTS = Frequency of urges to smoke; SUT=Strength of urges to smoke; HSI=Heaviness of smoking Index; s.d=standard deviation d.f=degrees of freedom – values provided are for the model and residual; IMD=Index of Multiple Deprivation

With the exception of age, ethnicity and IMD score, all significance testing between participant characteristics and measures of dependence conducted using Students t-test. Significance testing for age, ethnicity and IMD score conducted using Analysis of Variance (ANOVA).

### Multivariate modelling

The VIF and tolerance for the FUTS, SUTS and HSI measures were less than 10 and above 0.1; consequently, there was substantial collinearity between the measures of nicotine dependence. The associations between participant characteristics and smoking cessation are presented in Additional file 1: Table S2. Of variables that were significantly associated with both measure of dependence and smoking cessation, none adjusted the univariate odds ratio by ≥10% (Additional file 1: Table S3). Therefore, age, gender and study treatment group were retained in multivariate analyses by *a priori* justification. Age and IMD score were retained as continuous measures as there were no significant differences between the categorised and continuous variables. A linear relationship for the associations between SUTS and HSI measures and outcomes were found. In contrast, the relationship between FUTS and smoking cessation was non-linear (LRT p=0.0306; d.f=4). However, the findings have been reported as categorised and continuous variables for all measures of dependence to enable the comparison of findings with previous research [18,19]. The associations between the dependence measures and smoking cessation were not modified by age and gender.

### Association and predictive abilities of the measures of nicotine dependence for smoking cessation

The associations and predictive abilities of nicotine dependence measures for smoking cessation are presented in Table 2. As a continuous measure, every one-point increase in HSI was associated with a 16% (OR 0.84, 95% C.I 0.78-0.89; LRT p < 0.0001; d.f=1) reduction in the odds of smoking cessation six months after quitting. In contrast, every point increase in SUTS was associated with a non-significant 8% reduction in smoking cessation (OR 0.92, 95% C.I 0.84-1.02; LRT p=0.1000; d.f=1). As compared to those with the lowest FUTS (category 1), participants were initially more likely to report abstinence in the lower categories of FUTS (categories 2 and 3), but then as time spent with urges increased (category 5) participants were less likely to report cessation [(OR (95% C.I) for cessation in categories 2 and 5 of FUTS 1.35 (0.73-2.49) and 0.64 (0.33-1.22), respectively, LRT p for categories=0.0026); d.f=5]. The abilities to predict cessation for the HSI as a continuous variable [AUC (95% C.I)=0.63 (0.60-0.66)] and FUTS as a categorised variable [AUC (95% C.I)=0.63 (0.60-0.66)] measures were similar.

**Table 2:** **Associations and predictive abilities of nicotine dependence measures and smoking cessation**

**N**=**2,535**	**UNIVARIATE**		**MULTIVARIATE†**		**ROC ANALYSIS**	
	**OR (95% C.I)**	**P VALUE (d.f)‡**	**OR (95% C.I)**	**P VALUE (d.f)‡**	**AUC (95% C.I)**	P VALUE** (d.f)
FUTS CONTINUOUS	0.89 (0.81-0.97)	0.0067 (1)	0.88 (0.81-0.97)	0.0076 (1)	0.62 (0.59-0.64)	0.0769 (1)
SUTS CONTINUOUS	0.92 (0.84-1.01)	0.0747 (1)	0.92 (0.84-1.02)	0.1000 (1)	n/e	
HSI CONTINUOUS	0.88 (0.82-0.93)	<0.0001 (1)	0.84 (0.78-0.89)	<0.0001 (1)	0.63 (0.60-0.66)	
FUTS CATEGORISED						
1	1	<0.01 (5)	1	0.0026 (5)	0.63 (0.60-0.66)	0.2828 (1)
2	1.14 (0.64-2.02)		1.35 (0.73-2.49)			
3	1.19 (0.69-2.04)		1.39 (0.78-2.46)			
4	0.92 (0.54-1.59)		1.10 (0.62-1.96)			
5	0.55 (0.30-1.03)		0.64 (0.33-1.22)			
6	0.98 (0.51-1.90)		1.05 (0.52-2.13)			
SUTS CATEGORISED						
1	1	0.2693 (5)	1	0.3764 (5)	n/e	
2	0.98 (0.56-1.74)		1.16 (0.63-2.12)			
3	0.97 (0.59-1.61)		1.15 (0.67-1.97)			
4	0.99 (0.60-1.64)		1.09 (0.63-1.86)			
5	0.76 (0.43-1.32)		0.87 (0.48-1.57)			
6	0.57 (0.28-1.18)		0.70 (0.33-1.48)			
HSI CATEGORISED						
0	1	<0.01 (6)	1	<0.0001 (6)	0.64 (0.61-0.67)	
1	0.97 (0.60-1.57)		1.05 (0.63-1.74)			
2	1.01 (0.65-1.58)		0.98 (0.61-1.58)			
3	0.76 (0.49-1.17)		0.78 (0.49-1.23)			
4	0.76 (0.49-1.17)		0.70 (0.44-1.11)			
5	0.60 (0.38-0.97)		0.52 (0.31-0.86)			
6	0.37 (0.20-0.69)		0.26 (0.13-0.51)			

†adjusted for age, gender and treatment group [n=2,377 and n=2,404 for sample size retained in analyses for FUTS/SUTS and HSI, respectively]; ‡ Likelihood Ratio Test p value; **Chi-squared test p value; d.f=degrees of freedom

OR = Odds Ratio; C.I = confidence interval; FUTS = frequency of urges to smoke; SUTS = strength of urges to smoke; HSI = heaviness of smoking index; ROC = Receiver Operating Characteristic; AUC = Area under ROC curve; n/e = not entered into the ROC analysis model.

The results from the multiple imputation analyses are presented in Table 3. These findings from multivariate analyses were essentially the same as those from our primary analyses and FUTS and the HSI were significantly associated with cessation six months after participants quit smoking. The SUTS measure was not associated with abstinence. As a continuous measure, each point increase in the HSI score was association with a 15% reduction in the odds of smoking cessation at six months (OR 0.85 (95% C.I 0.79-0.92). The association between FUTS and smoking cessation was non-linear and, as compared to participants with the lowest score (category 1), those with higher FUTS (category 5) were 38% less likely to report abstinence (OR (95% C.I) for category 5 of FUTS 0.62 (0.27-1.39).

**Table 3:** **Multiple imputation analyses for associations of nicotine dependence measures and smoking cessation**

	**MULTIVARIATE†**		
	**OR (95% C.I)**	**P VALUE‡**	**Degrees of freedom**
FUTS CONTINUOUS	0.89 (0.80-0.99)	0.0271	Min: 94.1; Av: 115.4; Max: 162.3
SUTS CONTINUOUS	0.94 (0.86-1.04)	0.2334	Min: 103.1; Av: 126.7; Max: 166.9
HSI CONTINUOUS	0.85 (0.79-0.92)	<0.0001	Min: 101.8; Av: 122.8; Max: 164.0
FUTS CATEGORISED			
1	1	0.0186	Min: 56.6; Av: 90.9; Max: 165.0
2	1.31 (0.60-2.89)		
3	1.34 (0.64-2.82)		
4	1.09 (0.54-2.20)		
5	0.62 (0.27-1.39)		
6	0.98 (0.41-2.34)		
SUTS CATEGORISED			
1	1	0.6436	Min: 51.6; Av: 103.0; Max: 169.6
2	1.23 (0.62-2.42)		
3	1.21 (0.62-2.38)		
4	1.09 (0.60-2.01)		
5	0.96 (0.43-2.11)		
6	0.78 (0.36-1.71)		
HSI CATEGORISED			
0	1	0.0003	Min: 86.0; Av: 122.4; Max: 213.7
1	1.10 (0.63-1.95)		
2	0.95 (0.55-1.66)		
3	0.79 (0.48-1.32)		
4	0.83 (0.50-1.39)		
5	0.54 (0.31-0.95)		
6	0.25 (0.13-0.48)		

†adjusted for age, gender, treatment group; ‡ Joint Wald test Test p value; OR = Odds Ratio; C.I = confidence interval; FUTS = frequency of urges to smoke; SUTS = strength of urges to smoke; HSI = heaviness of smoking index; Min = minimum; Av = average; Max = maximum.

## Discussion

In a sample of smokers who sought smoking cessation support and participated in a trial set within a quitline, those with higher ratings of FUTS and the HSI were less likely to report smoking abstinence six months after quitting. For each point increase in HSI score participants were 16% less likely to remain abstinent. In addition, participants who experienced the greatest time spent with urges were 36% less likely to report cessation when compared to those with the least FUTS. Both FUTS and the HSI measures had similar predictive abilities for smoking cessation. The SUTS measure was not associated with abstinence in smokers who called a quitline for cessation support, which contrasts with the findings from previous research in other smoking populations [18,19].

### Strengths and Limitations

To our knowledge this is the largest study that has investigated the association between UTS and smoking cessation and the first such study conducted in motivated smokers who have sought quitline support . Furthermore, our findings were strengthened by the use of prospectively collected data, which enabled the temporal relationship between dependence measures and cessation outcomes to be investigated. Although only 1,354 (53.4%) participants provided follow-up data at six months our study used an intention to treat analysis and participants lost to follow up were assumed to be smokers. We explored the validity of this assumption by conducting, as a sensitivity investigation, a multiple imputation analysis that assumed outcome data were missing at random; there were no substantial differences between the findings from sensitivity and primary analyses.

Previous studies have investigated the single-item UTS measures as continuous variables [19,21,29,16] and assume their relationship with abstinence is linear. This is the first study to report analyses of these measures as both categorical and continuous variables. An unexpected finding was the non-linear relationship between FUTS and abstinence, which limited our ability to directly compare the relationship for HSI and FUTS measures to smoking cessation.

Our study collected data from the NHS smoking helpline as part of a RCT and the demography of participants was similar to that reported of people who set quit dates through community-based NHS stop smoking services [30]. This suggests that our findings may be representative of smokers who seek help with cessation generally. However, there was little ethnic diversity in the study population, so caution is needed when applying our findings to populations with higher proportions of ethnic minority groups.

Outcomes were not biochemically verified and the ascertainment of self-reported measures may have introduced recall and reporting bias resulting in the over-reporting of cessation [31]. However, the PORTSSS trial research staff were trained to encourage participants to provide honest accounts and any bias in reports of smoking status are likely to have been minimised [23,22].

Although we adjusted for the effects of confounding by the variables age, gender and treatment group from the PORTSSS trial, there are other factors that, in other studies, have been found to be associated with the success of quit attempts and for which data were not available. For example, the mental health status of smokers and baseline motivation to quit [27,32,33] have been reported as predictors of outcomes after quitting and such factors may also be associated with measures of nicotine dependence. Furthermore, we did not have data for other factors that may have influenced our findings such as other substance misuse and years of nicotine use. The potential for residual confounding, therefore, remains a possibility and could influence the observed associations in either direction and future research investigating the construct of nicotine dependence should ideally account for these variables.

### Comparison with previous research

Previous studies that investigated the association between single-item urges to smoke measures and cessation used data from the Smoking Toolkit Study (STS) and International Tobacco Control (ITC) four country survey; these studies involved a longitudinal design using repeat cross-sectional survey and included participants that were older, less nicotine dependent and more likely to be female than in our study [19,21,29,34]..

The first analysis from the STS found, as compared to the HSI, SUTS were most strongly associated with and had the greatest validity for predicting abstinence. Higher pre-quit SUTS was associated with a greater chance of relapse up to six months after quitting [18]. However, FUTS was not associated with abstinence. The second analysis from the STS investigated the association between smoking dependence and self-reported smoking status six months later [19]; greater SUTS were associated with a reduction in both abstinence and the chance of being a non-smoker six months later. In our study, both the HSI and FUTS measures were associated with and predictive of abstinence. SUTS, however, was not significantly associated with smoking cessation. Although the STS included a representative sample of English smokers the findings are not necessarily generalizable to other populations, such as smokers seeking cessation support. The contrasting findings may reflect differences in study populations, with our study including only motivated smokers who were seeking quitline support. However, it is difficult to think of a logical reason why, in one sample of smokers the perceived strength of urges should be predictive of cessation, whereas in the other, the frequency with which these were recalled was more important. It remains possible, therefore, that these divergent findings could arise due to instability and low reliability of the single-item measures used to measure the two constructs [35].

To date, there has only been one study that has compared the predictive ability of UTS with a validated measure of nicotine addiction [18]. Findings from the STS suggested that, based on the magnitude of association, SUTS had the greater ability to predict abstinence up to six months after quitting as compared to the HSI. Our study is the first to report the predictive abilities of UTS measures using ROC analyses; this enabled comparisons to be made between the categorical FUTS and continuous HSI variables, and enables the cut-off point of each measure to be evaluated for predicting outcomes. Both FUTS and the HSI measures were found to predict abstinence, which contrasts with the findings from previous research that included smokers who are not necessarily seeking help with cessation [18]. Our findings from ROC analyses were consistent with those from a study by Courvoisier and colleagues [13] who compared the abilities of several, non urges to smoke, nicotine dependence measures to predict abstinence 31 days after quitting, in smokers who accessed a cessation website. The authors reported that HSI had an ability to predict abstinence that was similar to our ROC findings which strengthens their validity. However, our study reports the predictive ability of the HSI for smoking cessation six months after quitting which conflicts with recent findings from the ITC four country survey [36]; Yong and colleagues reported that, although the HSI is predictive of short term relapse, it is not predictive of cessation beyond one month after quitting, and the contrasting findings may reflect differences in the populations between our study - that specifically included treatment seekers - and the ITC four country survey.

Most craving assessment tools focus on the strength of cravings experienced [35]. However, the severity of craving fluctuates throughout the course of a normal day and, as the SUTS measure ascertains the severity of cravings experienced by smokers in the past 24 hours, the lack of association between SUTS and abstinence may reflect the greater day-to-day instability and lower reliability of this single-item measure [35]. It has been suggested that the assessment of urge frequency may increase the yield of dependence measures for predicting cessation and this may have a greater utility when combined with other measures of cravings [35]. Furthermore, the findings for FUTS and the HSI in our study could reflect an overlap in the hypothetical construct of these measures; smokers who experience a greater urge frequency may smoke more over a typical day, although the analyses in the current study suggest there was no substantial collinearity amongst the measures of nicotine dependence.

Our study investigated the relationship between pre-quit measures of dependence and smoking cessation. A recent systematic review investigated the relationship between cravings and smoking cessation, and found the timing of craving assessment influences the success of smoking cessation [37]. Wray and colleagues found, as compared to studies where pre-quit cravings were assessed, studies that measured cravings after a quit attempt was initiated were more likely to detect an association for predicting abstinence. It is possible that cravings experienced by smokers before quitting are conceptually different to those after initiation of a quit attempt. Before quitting, smokers who are regularly smoking are likely to report a lower intensity of craving; therefore, pre-quit craving assessment, as in the current study, may be subject to floor effects reducing the strength of observed associations [37]. However, in our study the mean baseline FUTS, SUTS and HSI scores were at the mid-point of respective scales and the impact of floor effects is likely to be minimised.

## Conclusions

FUTS and the HSI were associated with and had a similar predictive ability for relapse six months after smokers made an attempt to quit. Single-item UTS measures are brief, easy to administer and may be pragmatic to measure the craving component of nicotine addiction. However, further research that tests the reproducibility, day-to-day stability and utility of combining the UTS as multi-item measures in other smoking cessation settings is required to provide a greater understanding of the role of these measures in the construct of nicotine addiction.

## Additional file

Additional file 1: Table S1. Comparison of baseline characteristics in participants who were followed up six months after quitting and those lost to follow up. **Table S2.** Associations between baseline characteristics and smoking cessation at six months after quitting. **Table S3.** The association between measures of nicotine dependence and smoking cessation at six months after quitting, and the change in odds ratio with addition of participant characteristics.

## Abbreviations

ANOVA: Analysis of variance
FTND: Fagerstrom test for Nicotine Dependence
FUTS: Frequency of urges to smoke
HSI: Heaviness of smoking index
IMD: Index of multiple deprivation
IQR: Interquartile range
LRT: Likelihood ratio test
PORTSSS: Trial Protocol for the Proactive Or Reactive Telephone Smoking CeSsation Support trial
PRIME: Theory Plans, Responses, Impulses, Motives and Evaluations
ROC: Receiver Operating Characteristic
SD: Standard deviation
SUTS: Strength of urges to smoke
UTS: Urges to smoke
VIF: Variance Inflation Factor

## References

[1] World Health Organization (1993). The ICD-10 Classification of Mental and Behavioural Disorders: Clinical Descriptions and Diagnostic Guidelines.

[2] American Psychiatric Association (1994). Diagnostic and Statistical Manual of Mental Disorders, Fourth Edition (DSM-IV).

[3] Doll R, Peto R, Boreham J, Sutherland I (2004). Mortality in relation to smoking: 50 years’ observations on male British doctors. Br Med J.

[4] Doll R, Peto R, Boreham J, Sutherland I (2005). Mortality from cancer in relation to smoking: 50 years observations on British doctors. Br J Cancer.

[5] West R (2004). Assessment of dependence and motivation to stop smoking. Br Med J.

[6] Srivastava P, Currie GP, Britton J (2006). Smoking cessation. BMJ.

[7] Molyneux A (2004). Nicotine replacement therapy. Br Med J.

[8] Coleman T (2004). Use of simple advice and behavioural support. Br Med J.

[9] Bock G, Goode J (2006). Understanding nicotine and tobacco addiction.

[10] Tobacco Advisory Group of The Royal College of Physicians (2000). Nicotine Addiction in Britain.

[11] Heatherton TF, Kozlowski LT, Frecker RC, Fagerstrom KO (1991). The fagerstrom test for nicotine dependence: a revision of the fagerstrom tolerance questionnaire. Br J Addict.

[12] Kozlowski LT, Porter CQ, Orleans CT, Pope MA, Heatherton T (1994). Predicting smoking cessation with self-reported measures of nicotine dependence: FTQ, FTND, and HSI. Drug Alcohol Depend.

[13] Courvoisier DS, Etter JF (2010). Comparing the predictive validity of five cigarette dependence questionnaires. Drug Alcohol Depend.

[14] American Psychiatric Association (2013). Diagnostic and statistical manual of mental disorders.

[15] West R (2009). The multiple facets of cigarette addiction and what they mean for encouraging and helping smokers to stop. COPD.

[16] West RJ, Hajek P, Belcher M (1989). Severity of withdrawal symptoms as a predictor of outcome of an attempt to quit smoking. Psychol Med.

[17] West R, Hajek P (2004). Evaluation of the mood and physical symptoms scale (MPSS) to assess cigarette withdrawal. Psychopharmacology (Berl).

[18] Fidler JA, Shahab L, West R (2011). Strength of urges to smoke as a measure of severity of cigarette dependence: comparison with the fagerstrom test for nicotine dependence and its components. Addiction.

[19] Fidler JA, West R (2011). Enjoyment of smoking and urges to smoke as predictors of attempts and success of attempts to stop smoking: a longitudinal study. Drug Alcohol Depend.

[20] Fidler JA, Shahab L, West O, Jarvis MJ, McEwen A, Stapleton JA (2011). ‘The smoking toolkit study’: a national study of smoking and smoking cessation in England. BMC Public Health.

[21] Herd N, Borland R, Hyland A (2009). Predictors of smoking relapse by duration of abstinence: findings from the international tobacco control (ITC) four country survey. Addiction.

[22] Coleman T, McEwen A, Bauld L, Ferguson J, Lorgelly P, Lewis S (2009). Protocol for the proactive or reactive telephone smoking CeSsation support (PORTSSS) trial. Trials.

[23] Ferguson J, Docherty G, Bauld L, Lewis S, Lorgelly P, Boyd KA (2012). Effect of offering different levels of support and free nicotine replacement therapy via an English national telephone quitline: randomised controlled trial. Br Med J.

[24] UK Statistics Authority. Index of Multiple Deprivation (IMD). Office for National Statistics. 2007. http://data.gov.uk/dataset/index_of_multiple_deprivation_imd_2007.

[25] West R, Hajek P, Stead L, Stapleton J (2005). Outcome criteria in smoking cessation trials: proposal for a common standard. Addiction.

[26] Hamilton LC (2006). Statistics with Stata.

[27] Zhou X, Nonnemaker J, Sherrill B, Gilsenan AW, Coste F, West R (2009). Attempts to quit smoking and relapse: factors associated with success or failure from the ATTEMPT cohort study. Addict Behav.

[28] Altman DG, Bland JM (1994). Diagnostic tests 3: receiver operating characteristic plots. Br Med J.

[29] Shiffman S, Engberg JB, Paty JA, Perz WG, Gnys M, Kassel JD (1997). A day at a time: predicting smoking lapse from daily urge. J Abnorm Psychol.

[30] Health & Social Care Information Centre (2013). Statistics on NHS Stop Smoking Services: England, April 2012 – March 2013.

[31] Gorber SC, Schofield-Hurwitz S, Hardt J, Levasseur G, Tremblay M (2009). The accuracy of self-reported smoking: a systematic review of the relationship between self-reported and cotinine-assessed smoking status. Nicotine Tob Res.

[32] Osler M, Prescott E (1998). Psychosocial, behavioural, and health determinants of successful smoking cessation: a longitudinal study of Danish adults. Tob Control.

[33] Lee CW, Kahende J (2007). Factors associated with successful smoking cessation in the United States, 2000. Am J Public Health.

[34] Thompson ME, Fong GT, Hammond D, Boudreau C, Driezen P, Hyland A (2006). Methods of the international tobacco control (ITC) four country survey. Tob Control.

[35] Tiffany ST, Wray JM (2012). The clinical significance of drug craving. Ann N Y Acad Sci.

[36] Yong HH, Borland R, Balmford J, Hyland A, O’Connor RJ, Thompson ME et al. Heaviness of Smoking Predicts Smoking Relapse Only in the First Weeks of a Quit Attempt: Findings From the International Tobacco Control Four-Country Survey. Nicotine & Tobacco Research. 2013;24:[Epub ahead of print]. 10.1093/ntr/ntt165 PMC3954420 24158228

[37] Wray JM, Gass JC, Tiffany ST (2013). A systematic review of the relationships between craving and smoking cessation. Nicotine Tob Res.

