# Supplementary material for: Do cravings predict smoking cessation in smokers calling a national quit line: secondary analyses from a randomised trial for the utility of ‘urges to smoke’ measures
Source: Subst Abuse Treat Prev Policy. 2015 Apr 14;10:15. doi: 10.1186/s13011-015-0011-8 (PMC4414292; doi:10.1186/s13011-015-0011-8)
Supplement: Additional file 1: Table S1. — Comparison of baseline characteristics in participants who were followed up six months after quitting and those lost to follow up. Table S2. Associations between baseline characteristics and smoking cessation at six months after quitting. Table S3. The association between measures of nicotine dependence and smoking cessation at six months after quitting, and the change in odds ratio with addition of participant characteristics. [file 13011_2015_11_MOESM1_ESM.docx]

**Additional file 1: Table S1: Comparison of baseline characteristics in participants who were followed up six months after quitting and those lost to follow up**

|  | Followed up  (n=1,354) | Lost to follow up  (n=1,181) | P value** |
| --- | --- | --- | --- |
| Age†* | 40 (30-53) | 35 (25-46) | <0.0001 |
| Gender*  Male  Female | 606 (44.8)  721 (53.3) | 531 (45.0)  634 (53.7) | 0.461 |
| Ethnicity  White  Black/mixed  Asian/mixed  Other | 1,210 (89.4)  55 (4.1)  50 (3.7)  39 (2.9) | 1,071 (90.7)  41 (3.5)  49 (4.2)  20 (1.7) | 0.183 |
| IMD score† | 22.1 (13.0-36.7) | 24.0 (14.1-38.3) | 0.0382 |
| Smoker in same household*  No  Yes | 871 (64.3)  477 (35.2) | 731 (61.9)  446 (37.8) | 0.391 |
| Trial intervention  Reactive support without NRT  Reactive support with NRT  Proactive support without NRT  Proactive support with NRT | 353 (26.1)  333 (24.6)  331 (24.5)  337 (24.9) | 280 (23.7)  305 (25.8)  301 (25.5)  295 (25.0) | 0.559 |
| FUTS‡* | 3.5 (1.2) | 3.6 (1.2) | 0.0531 |
| SUTS‡* | 3.5 (1.1) | 3.6 (1.2) | 0.1200 |
| HSI ‡* | 3.1 (1.6) | 3.3 (1.6) | 0.0042 |

IMD = Index of multiple Deprivation; FUTS = Frequency of Urges to Smoke; SUTS = Strength of Urges to Smoke; HSI = Heaviness of Smoking Index

† = Median (IQR); ‡ = Mean (SD); all other values = n (column %)

** Statistical tests used = Chi-squared test (Gender, ethnicity, smoker in same household, trial intervention); Mann-Whitney (Age, IMD score); Students t-test (FUTS: d.f=2482; SUTS: d.f=2482; HSI: d.f=2509)

* Missing data in participants followed up: n= 38 (age) n=27 (gender), n=6 (smoker in same household), n=27 (FUTS), n=36 (SUTS), n=15 (HSI)

* Missing data in participants lost to follow up: n= 28 (age) n=16 (gender), n=4 (smoker in same household), n=24 (FUTS), n=15 (SUTS), n=9 (HSI)

**Additional file 1: Table S2: Associations between baseline characteristics and smoking cessation at six months after quitting**

|  | Smoking cessation | |
| --- | --- | --- |
|  | OR (95% C.I) | P value (d.f)* |
| Age (years) | 1.02 (1.01-1.03) | <0.001 (1) |
| Gender  Male  Female | 1  0.75 (0.61-0.92) | <0.01 (1) |
| Ethnicity  White  Black/mixed  Asian/mixed  Other | 1  1.10 (0.66-1.84)  1.59 (1.00-2.52)  0.60 (0.27-1.33) | 0.1269 (3) |
| IMD score | 1.00 (0.99-1.00) | 0.121 (1) |
| Smoker in same household  No  Yes | 1  0.85 (0.69-1.06) | 0.1426 (1) |
| Free prescription entitlement  No  Yes | 1  0.79 (0.64-0.96) | <0.05 (1) |
| Quit attempt in last 12 months  No  Yes | 1  0.73 (0.59-0.90) | <0.01 (1) |
| Cessation support used last quit attempt  No  Yes | 1  0.70 (0.55-0.88) | <0.01 (1) |
| Current use of cessation support  No  Yes | 1  1.70 (1.34-2.15) | <0.001 (1) |
| Trial intervention  Reactive support without NRT  Reactive support with NRT  Proactive support without NRT  Proactive support with NRT | 1  0.87 (0.65-1.15)  0.97 (0.74-1.28)  0.78 (0.59-1.05) | 0.3211 (3) |

NRT = Nicotine Replacement Therapy

* Univariate logistic regression Wald Test P value; d.f = degrees of freedom

Additional file 1: Table S3: The association between measures of nicotine dependence and smoking cessation at six months after quitting, and the change in odds ratio with addition of participant characteristics

|  | LONG-TERM SMOKING CESSATION | | |
| --- | --- | --- | --- |
|  | FUTS | SUTS | HSI |
| UNIVARIATE ODDS RATIO (95% CI) | 0.89  (0.81-0.97) | 0.92  (0.84-1.01) | 0.88  (0.82-0.93) |
|  |  |  |  |
| VARIABLE | OR (≥ 10% CHANGE IN ODDS RATIO) | | |
| Age (years) | 0.89 (N) | 0.93 (N) | 0.84 (N) |
| Gender | 0.88 (N) | 0.92 (N) | 0.88 (N) |
| Ethnicity | 0.89 (N) | 0.92 (N) | 0.88 (N) |
| IMD score | 0.89 (N) | 0.92 (N) | 0.88 (N) |
| Age finished education (years) | 0.88 (N) | 0.92 (N) | 0.86 (N) |
| Smoker in same household | 0.89 (N) | 0.92 (N) | 0.88 (N) |
| Free NHS prescriptions | 0.90 (N) | 0.94 (N) | 0.88 (N) |
| Quit attempt in last 12 months | 0.89 (N) | 0.93 (N) | 0.87 (N) |
| Cessation support used last quit attempt | 0.89 (N) | 0.93 (N) | 0.88 (N) |
| Current use of cessation support | 0.88 (N) | 0.92 (N) | 0.87 (N) |
| Allocated support in study | 0.89 (N) | 0.92 (N) | 0.88 (N) |
| Allocated NRT in study | 0.89 (N) | 0.92 (N) | 0.88 (N) |
| FUTS | N/A | 0.97 (N) | 0.90 (N) |
| SUTS | 0.88 (N) | N/A | 0.88 (N) |
| HSI | 0.94 (N) | 0.97 (N) | N/A |

FUTS = frequency of urges to smoke; SUTS = strength of urges to smoke; HSI = heaviness of smoking index; NRT = Nicotine Replacement Therapy

(N) = Not associated with ≥ 10 change in univariate odds ratio

N/A = Not applicable
